# Before platelets: the production of platelet-activating factor during growth and stress in a basal marine organism

**DOI:** 10.1098/rspb.2018.1307

**Published:** 2018-08-15

**Authors:** Ines Galtier d'Auriac, Robert A. Quinn, Heather Maughan, Louis-Felix Nothias, Mark Little, Clifford A. Kapono, Ana Cobian, Brandon T. Reyes, Kevin Green, Steven D. Quistad, Matthieu Leray, Jennifer E. Smith, Pieter C. Dorrestein, Forest Rohwer, Dimitri D. Deheyn, Aaron C. Hartmann

**Affiliations:** 1 Department of Biology, San Diego State University, San Diego, CA 92182-4614, USA; 2 Skaggs School of Pharmacy and Pharmaceutical Sciences, University of California, San Diego, CA 92093, USA; 3 Scripps Institution of Oceanography, University of California, San Diego, CA 92093, USA; 4 Ronin Institute, NJ 07043, USA; 5 Laboratoire de Génétique de l'Evolution (LGE), Institute of Chemistry, Biology, and Innovation, ESPCI ParisTech/CNRS UMR 8231/PSL Research University, Paris, France; 6 Smithsonian Tropical Research Institute, Smithsonian Institution, Panama City, Republic of Panama; 7 National Museum of Natural History, Smithsonian Institution, Washington, DC, USA

**Keywords:** coral reef ecology, phospholipids, metabolomics, platelet-activating factor

## Abstract

Corals and humans represent two extremely disparate metazoan lineages and are therefore useful for comparative evolutionary studies. Two lipid-based molecules that are central to human immunity, platelet-activating factor (PAF) and Lyso-PAF were recently identified in scleractinian corals. To identify processes in corals that involve these molecules, PAF and Lyso-PAF biosynthesis was quantified in conditions known to stimulate PAF production in mammals (tissue growth and exposure to elevated levels of ultraviolet light) and in conditions unique to corals (competing with neighbouring colonies over benthic space). Similar to observations in mammals, PAF production was higher in regions of active tissue growth and increased when corals were exposed to elevated levels of ultraviolet light. PAF production also increased when corals were attacked by the stinging cells of a neighbouring colony, though only the attacked coral exhibited an increase in PAF. This reaction was observed in adjacent areas of the colony, indicating that this response is coordinated across multiple polyps including those not directly subject to the stress. PAF and Lyso-PAF are involved in coral stress responses that are both shared with mammals and unique to the ecology of cnidarians.

## 1. Introduction

Cnidarians (corals, jellyfish and anemones) are probably among the first metazoans [1], making them an ideal system to investigate how the first immune systems may have been structured. Cnidarians do not produce the diversity of cell types central to immunity in more recently evolved lineages such as mammals (e.g. T- and B-cells), but Cnidaria and Chordata (including mammals) share many immune processes and components present in the latter [2–8]. For example, enzymes involved in the biosynthesis and modification of platelet-activating factor (PAF) [9], a phospholipid-derived signalling molecule often studied in humans, are also produced by corals [10]. Consistent with the presence of PAF-related genes in coral genomes, PAF and its precursor Lyso-PAF were recently detected in coral metabolomes, and the latter was among the most abundant molecules detected [10]. The presence of these immune modulators in corals begs the question of whether PAF and Lyso-PAF are produced in response to similar environmental stimuli, which would suggest conserved processes, or whether PAF is produced in processes specific to the ecology of reef-building corals.

Diverse taxonomic groups produce PAF, including mammals, cnidarians, protozoans, yeasts, plants and bacteria. In mammals, PAF is involved in tissue growth [11–13] and the initiation of numerous immune responses, including coagulation, inflammation, and immune cell proliferation and migration to lesion sites [9,14–18]. Coral immune systems exhibit many similar processes, including coagulation, immune cell migration to lesions and inflammatory reactions [19–21], though PAF has rarely been implicated [10]. These processes are carried out in corals via the expression of a large repertoire of immune genes, including those that may be related to pathways involving PAF [4,6,8]. While PAF has been well studied, the full diversity of its functions remains unclear, in part owing to the complexity of processes in which it is involved. For example, PAF acts as an immunosuppressor (as opposed to an immune inducer) in response to tissue damage from excessive ultraviolet radiation (UVR) exposure [14,22]. By quantifying the production of PAF in corals under different stimuli, this study offers insights into the roles of this molecule in one of the most ancient immune systems on the planet.

To probe the potential involvement of PAF in growth and stress responses in corals, the molecule was quantified during accelerated tissue growth, during conditions known to induce cellular damage and when one coral was attacking another coral. Active tissue growth and the induction of cellular damage were generated by modifying the light environment experienced by the coral. Coral growth increases with the amount of visible white light (though excessive white light inhibits growth) [23], while UVR exposure causes cellular damage [24], much as it does in humans. Corals attack one another during competition for space on and above the benthos, often using their mesenterial filaments, a mass of long tentacle-like organs normally held within the polyp stomach that can be protruded from the mouth. Coral tentacles and mesenteries contain many cnidocyte cells that release a harpoon-like microstructure (nematocyst) that injects venom directly into their target organism. Nematocyst venom is replete with phospholipase A2 (PLA2), the enzyme that catalyses the formation of PAF [25]. Therefore, we hypothesized that PLA2-stimulated production of PAF arises when corals are attacked by neighbouring colonies. Through laboratory experiments and field observations we found that PAF biosynthesis increased when corals were exposed to elevated levels of UVR, in the growing region of coral tissue, and when corals were being physically attacked by other corals. We conclude that corals produce PAF during multiple processes, including when responding to a broad stressor (UVR exposure), growing new tissue and experiencing an acute stress (direct physical attack).

## 2. Materials and methods

Experiments were conducted using the branching Pacific coral *Acropora yongei* that was maintained in a flow-through culture system at Scripps Institution of Oceanography (SIO; La Jolla, CA). The original collection consisted of a single fragment that was grown, fragmented, and maintained at SIO and the Birch Aquarium at Scripps (La Jolla, CA). Thus, all experiments were carried out using a putative single genotype. ‘Colonies’ refer to larger pieces of this genet that contained multiple branches.

### (a) Growth experiment

To test whether Lyso-PAF and PAF production changes during coral growth, a pre-experiment was conducted to measure the difference in linear extension of branches between shaded and non-shaded *A. yongei* colonies. *Acropora yongei* colonies were placed into four aquaria. The corals were acclimated to the aquarium light conditions for 4 days prior to the experiment using a Giesemann System 260 programmable light system with T5 fluorescent lights (Geismann aquaristic, Germany). After acclimation, two of the aquaria were covered with a 2 mm thick semi-porous screen to reduce the amount of light reaching the coral (henceforth referred to as the ‘shaded/20% UVR treatment’). Two aquaria were not modified and served as controls for the coral growth that occurred under the laboratory light conditions (henceforth referred to as the ‘non-shaded/20% UVR treatment’). Light concentrations were measured throughout the experiments using Hobo light and temperature loggers (Onset Computer Corporation, MA, USA). The linear extension of coral branch tips, the site of growth, was measured using a ruler prior to the start of the experiment and after 12 days. Shading decreased growth of branch tips by 77% relative to non-shaded corals (shaded = mean of 0.12 cm linear extension, non-shaded = mean of 0.35 cm linear extension; *n* = 25 branch tips, *p* = 0.001), confirming that shading reduced coral growth in our experimental set-up and that this manipulation could be used to determine whether Lyso-PAF and PAF production changes during growth.

The shaded and non-shaded corals were allowed to acclimate for one week; then the experimental set-up of the pre-experiment was repeated. Corals were sampled after 6 days by cutting two samples from each coral branch: the growing tips of each colony were sampled as the top-most cm of a branch, while the non-growing bases were sampled as 1 cm below the bottom of the tip (i.e. the third cm of a branch). In each light condition, 25 tips and 25 paired bases were sampled to measure PAF abundance in growing tissues versus non-growing tissues and at different growth rates. Measuring Lyso-PAF and PAF in the non-growing bases controlled for any non-growth-related changes that occurred due to higher light (e.g. differences in UVR exposure). Immediately after cutting, samples were snap-frozen in liquid nitrogen. Molecules were later extracted by placing the frozen sample in 2 ml of liquid chromatography tandem mass spectrometry (LC-MS/MS) grade 70% methanol for 3 days at 4°C. The resulting extracts were then stored at −80°C.

### (b) Ultraviolet radiation experiment

To isolate the effects of UVR exposure (a presumed stress) from growth (normal physiology), we conducted a subsequent experiment examining PAF content in the bases of corals exposed to increased UVR. Colonies from the non-shaded treatment that were not sampled during the growth experiment were exposed to increased UVR. The programmability of the Geismann light includes the ability to change the level of UVR. For the experiment, UVR was increased from 20% to 80% (20% was used throughout the acclimation period and growth experiments) over three *A. yongei* colonies in each of two aquaria. This additional treatment is referred to as the ‘non-shaded/80% UVR treatment’. After 24 h, 11 bases were sampled. Samples were stored and extracted as indicated above for the growth experiment.

### (c) Time-course experiment

At Birch Aquarium at Scripps (La Jolla, CA), five *A. yongei* and five *Pocillopora damicornis* aquarium corals were moved from a reserve aquarium, where they coexisted with a mixed reef community, to a new aquarium in the same flow-through seawater system. After 4 h of acclimation, five pairs of *A. yongei* and *P. damicornis* colonies were placed in close proximity to one another (distanced by a few millimetres), but not in direct physical contact. The proximity necessary to stimulate an interaction between two colonies was determined in previous manipulations (see the electronic supplementary material, movie S1 for example). *Acropora yongei* colonies were consistently observed to attack *P. damicornis* colonies with their mesenterial filaments, while *P. damicornis* colonies did not exhibit any visually detectable defence response. The interaction zone was defined as the area closest to the neighbouring colony and, on the *P. damicornis*, the area attacked by the mesenteries of the *A. yongei*. Coral tip samples were taken at the interaction zone and at the non-interaction zone after 1, 5, 10 and 15 min of mesenterial attack (*n* = 5/area × time). Non-interaction zones were branch tips of a colony away from the site of interaction and served as a control for the change in molecules produced at sites of coral–coral interactions. Each tip sample was immediately placed in 2 ml of LC-MS/MS grade 70% methanol for 3 days at 4°C for lipid extraction. The resulting extracts were then stored at −80°C.

### (d) Coral interactions *in situ*

*Acropora cervicornis*, *Porites porites* and *Madracis mirabilis* in close proximity to one another or alone were sampled from five Salt Creek reefs around Bocas del Toro, Panama (9.280774, −82.104946). For the molecular three-dimensional (3D) cartography of PAF in coral interactions, five contiguous 1 cm fragments were sampled from each coral branch starting from the growing tips. Samples were extracted in LC-MS/MS grade 70% methanol and the extracts were stored at −80°C.

### (e) Sample preparation for metabolomics analysis and batch design

After being weighed, the lipid extracts were resuspended in 70% methanol and added to a 96-well plate with 70% methanol blanks at the end of each row (i.e. every 11 samples). A quality control standard mix was also analysed in three intervals throughout the run of each plate. Each sample well contained 150 µl of sample and 10 µl of 0.01 µM amitriptyline as a standard that was used to account for variation in ionization efficiency among samples during analysis.

### (f) Mass spectrometry analysis

Ultra performance liquid chromatography tandem mass spectrometry (UPLC-MS/MS) mass spectrometry was carried out using an UltiMate 3000 UPLC system (Thermo Scientific), controlled by the Chromeleon software (Thermo Scientific) coupled to a maXis quadrupole time of flight mass spectrometer (Bruker Daltonics), using the Otof Control and Hystar software packages (Bruker Daltonics) and equipped with an electrospray ionization (ESI) source. UPLC conditions of analysis were as follows: 1.7-µm C18 (50 × 2.1-mm) ultra-high performance liquid chromatography column (Phenomenex); column temperature, 40°C; flow rate, 0.5 ml min^−1^; mobile phase A, 98% water/2% acetonitrile/0.1% formic acid (vol/vol); mobile phase B, 98% acetonitrile/2% water/0.1% formic acid (vol/vol). A linear gradient was used for the chromatographic separation: 0–2 min, 0–20% B; 2–8 min, 20–100% B; 8–9 min, 100–100% B; 9–10 min, 0% B. MS spectra were acquired in a positive ion mode in the mass range *m/z* 50–2000. Instrument parameters were set as follows: nebulizer gas (nitrogen) pressure, 2 bar; capillary voltage, 4500 V; ion source temperature, 180°C; dry gas flow, 9 l min^−1^; spectra rate acquisition, 10 spectra s^−1^. MS/MS fragmentation of the 10 most intense selected ions per spectrum was performed using ramped collision-induced dissociation energy, ranged from 35 eV for +1 ions and 25 eV for +2 ions in the collision cell with automatic exclusion of the spectra after three counts for 10 s.

### (g) Analysis of LC-MS/MS data

The Bruker^®^ Daltonics Find Molecular Features algorithm was used to identify molecular features in each sample (Bruker^®^ Data Analysis software v. 4.2 build 4.2.395.0) using the following parameters: a signal-to-noise threshold of 5, correlation coefficient threshold of 0.7, a minimum compound length of 8 spectra and a smoothing width of 2. The number of detected molecular features in each experiment were: 2150 (UVR experiment), 5769 (timeline experiment), 8700 (*in situ* interactions) and 6500 (molecular cartography of *in situ* interactions). Peak area was normalized to the total abundance of all molecules in the sample. Spectral data were converted to mzXML using CompassXport (Bruker, Daltonics, Bremen, Germany) and files were uploaded to the Global Natural Products Social Molecular Network (GNPS) database [26] and deposited to Massive ID under the reference MSV000080662. Molecular networks of LC-MS/MS data were created using the GNPS data analysis workflow (https://gnps.ucsd.edu/ProteoSAFe/status.jsp?task=42443130e8a5451dbad8963736b803d7). The default parameters were used for molecular networks except for the following: a precursor mass tolerance of 0.1 Da and a MS/MS fragment ion tolerance of 0.1 Da to create consensus spectra. Molecular networks were visualized using Cytoscape 3.3.0 [27] (electronic supplementary material, figure S5). The MS/MS spectra were searched against GNPS's spectral libraries and others including Massbank, ReSpect, HMDB and NIST14. Detected features were considered a match to known spectra when they had a cosine score above 0.7, at least six matched peaks and less than 20 ppm of relative error.

### (h) Statistics

For all experiments, the area under the curve of ‘PAF C16’ (*m/z* 524.371) and ‘Lyso-PAF C16’ (*m/z* 482.361) was calculated manually using the Data Analysis software (Bruker Corporation, MA, USA). The abundances of PAF and Lyso-PAF were normalized to a standard (amitriptyline) abundance per sample as well as the individual mass per sample. Samples from the time-course experiment were normalized to the dry mass of the lipid extract. Pairwise comparisons for the UVR and field interaction experiments were made on raw data using a *t*-test for normally distributed data and the non-parametric Mann–Whitney *U*-test for non-normally distributed data. Datasets with more than two groups were analysed with analysis of variance (ANOVA) following a log transformation in order to achieve normality (e.g. *p* < 0.001 for a Shapiro test using raw PAF values and *p* = 0.5 following log transformation). ANOVA was used to compare the two main factors in the growth experiment: location (tip versus base) and light intensity (non-shaded versus shaded). A Tukey's honestly significant difference test was used to make pairwise comparisons of the four treatment groups. PAF abundance across discrete sampling points through time were compared with an ANOVA and differences between interacting and non-interacting branches were compared at each time point with a Mann–Whitney *U*-test.

### (i) Molecular three-dimensional cartography generation

All corals pictures were taken with a GoPro underwater camera, merged into a 3D model (coral interactions and controls) using Autodesk 123D Catch, and then duplicated into four models. A 3D model was created and exported as an .stl file and then imported into MeshLab v.1.3.3 to determine the coordinates corresponding to each sample (five points per coral). An MS/MS feature file was generated using the Optimus workflow (v. 2016.7.21, Alexandrov Team, EMBL Heidelberg) as described in [28], with a noise threshold intensity of 10 000. Peaks were identified as molecular features when their intensity was at least three times higher than their maximum intensity in any blank. The samples were associated to the corresponding 3D coordinates using the Optimus workflow to create a csv file. The 3D model and the corresponding MS/MS data were visualized using ‘ili toolbox (https://github.com/MolecularCartography/ili). Graphing and statistical analyses were performed using GraphPad Prism 6 (GraphPad Software, San Diego, CA). The midline represents the median. The 3D model and csv file were uploaded as part of the MSV000080662 submission to GNPS.

### (j) Bioinformatic identification of enzyme homologues involved in platelet-activating factor mode of action

Human protein sequences of the PAF receptor (GI: 298581) and protein kinase C (GI: 4506067) were used with tBLASTn to query a coral transcript database generated from transcripts sequenced and described earlier [10].

## 3. Results

### (a) Platelet-activating factor production is higher in growing regions of corals

**Figure 1.**
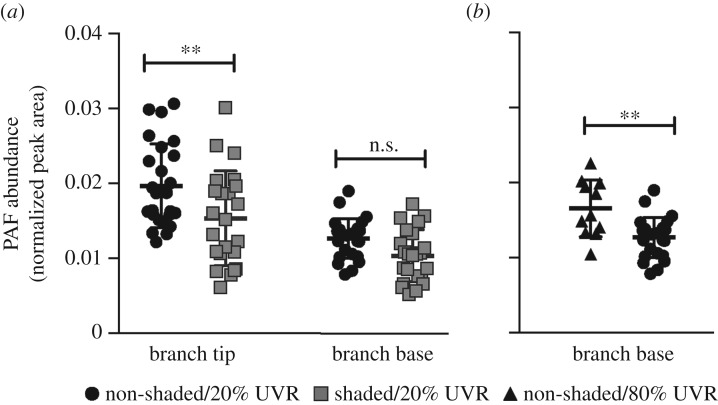
Normalized abundances of platelet-activating factor (PAF; mean ± s.d.) in *Acropora yongei* corals depending on the location on (*a*) a branch (tip or base) and light conditions (shaded and non-shaded). To test the effect of tissue growth on PAF production, growing branch tips were compared to non-growing branch bases grown under non-shaded/20% UVR (*n* = 25 for both, black circles) and shaded/20% UVR (*n* = 23 for both, grey squares) conditions (to create high and low growth in the tips, respectively). To test the effect of elevated UVR exposure on PAF production, UVR was increased by 4X and the PAF content was (*b*) compared between non-growing bases grown under non-shaded/80% UVR (*n* = 11, black triangles) and the non-shaded/20% UVR conditions from (*a*). Data in (*a*) were log-transformed and compared with an analysis of variance. Data in (*b*) were compared with a Mann–Whitney *U*-test. Asterisks denote the level of significance (n.s., **p < 0.01).

Given the role of PAF in mammalian tissue growth, PAF production was sampled during active coral tissue growth by sampling the growing region (tip) and non-growing region (base) under non-shaded and shaded light regimes (higher light exposure is associated with faster growth). PAF content was greater in coral branch tips compared to coral branch bases (*p* < 0.001) and in non-shaded colonies compared to shaded colonies (*p* < 0.001; figure 1*a*). Non-shaded branch tips had 1.3 times higher PAF levels than shaded branch tips (*p* = 0.01), though non-shaded bases had only marginally different PAF content relative to shaded bases (*p* = 0.06). The significant difference in PAF in growing tips at different light intensities and the marginal difference in the non-growing bases suggest that PAF production is associated with growth, an observation that has also been made in growing mammalian tissues [11–13]. However, PAF production (e.g. in branch bases) may also be responding to differences in light intensity, perhaps outside the visible light spectrum.

### (b) Platelet-activating factor production increases when corals are exposed to elevated levels of ultraviolet radiation

Previous investigations of PAF production in mice and humans showed that PAF abundance is elevated when skin tissue is exposed to elevated levels of UVR [14,22]. To determine if UVR also leads to PAF production in corals, *A. yongei* colonies from the growth experiment were exposed to a higher level of UVR intensity and the PAF content of the non-growing branch bases was compared before and after the increases in UVR. The PAF content of branch bases was approximately 1.3 times greater in the non-shaded/80% UVR treatment compared to the non-shaded/20% UVR treatment (*p* = 0.002; figure 1*b*). These results suggest that, similarly to humans, experiencing elevated UVR radiation, and probably the cellular stress induced by it, leads to increased production of PAF in corals.

### (c) Platelet-activating factor production is higher when corals are attacked by stinging cells of neighbouring corals

It was recently reported that PAF production is higher in damaged coral tissue next to competitors [10]. To determine whether PAF increases in damaged tissues due to being attacked by a neighbouring colony, we used a model of experimentally induced coral interactions in which *A. yongei* rapidly extruded its mesenteries onto neighbouring *Pocillopora damicornis* colonies, resulting in tissue damage and eventual death in the latter (electronic supplementary material, movie S1).

**Figure 2.**
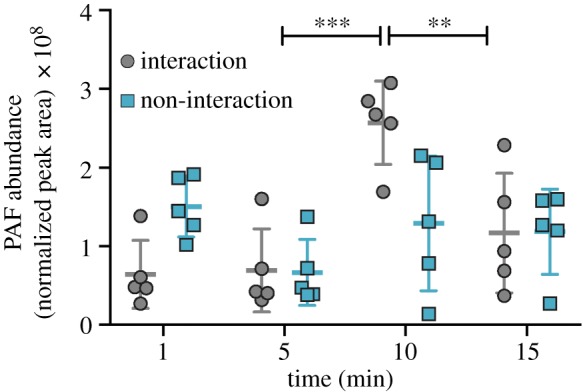
Normalized abundances (mean ± s.d.) of platelet-activating factor (PAF) in *Pocillopora damicornis* branches that were interacting with *Acropora yongei* (grey circles, *p* < 0.001 though time) and not interacting with *A. yongei* (teal squares, *p* = 0.09). *Pocillopora damicornis* branches were collected 1, 5, 10 and 15 min after the attack began. Differences in PAF abundances between the interaction or non-interaction samples were compared across time with ANOVA and pairwise comparisons were made at each time point using a Mann–Whitney *U*-test owing to the non-normality of the data. Asterisks denote the level of significance (**p < 0.01, ***p < 0.001).

A time-course experiment was conducted to directly test whether PAF production increases in response to mesenterial attack by *A. yongei* on *P. damicornis*. In aquaria with circulating seawater, colonies of the two species were placed adjacent to each other. After 4 h, *A. yongei* extruded its mesenteries and began making contact with *P. damicornis*. Sampling was then carried out 1, 5, 10 and 15 min after the start of the mesenterial attack. In *P. damicornis*, the attacked coral, PAF content changed over the course of the experiment in the branch that was directly attacked (*p* < 0.01, one-way ANOVA of discrete time points). PAF abundance remained unchanged after 1 and 5 min of mesenterial contact (i.e. interaction; figure 2; *p* = 0.28; unpaired *t*-test) but increased between 5 and 10 min of interaction (figure 2; *p* < 0.001) and then decreased between 10 and 15 min of interaction (figure 2; *p =* 0.01). By contrast, Lyso-PAF abundances remained unchanged in up to 10 min of interaction (electronic supplementary material, figure S1; *p* = 0.08) and then increased from 10 to 15 min (*p =* 0.02). PAF abundances did not change significantly over time in branches of the same *P. damicornis* colonies that were not interacting with *A. yongei* (*p* = 0.09, one-way ANOVA of discrete time points). Similar results were obtained for measurements of Lyso-PAF in control fragments (electronic supplementary material, figure S1; *p* = 0.07). Observations from the coral interactions revealed that *P. damicornis* tissue was degraded, bleached and receded from the areas of physical attack, while the *A. yongei* tissue was visually unaffected (electronic supplementary material, movie S1). Consistently, PAF and Lyso-PAF productions were stable in *A. yongei* during competition (electronic supplementary material, figure S2 and S3, respectively; *p* = 0.23 and *p* = 0.35, respectively).

### (d) Platelet-activating factor production during coral interactions on the reef

**Figure 3.**
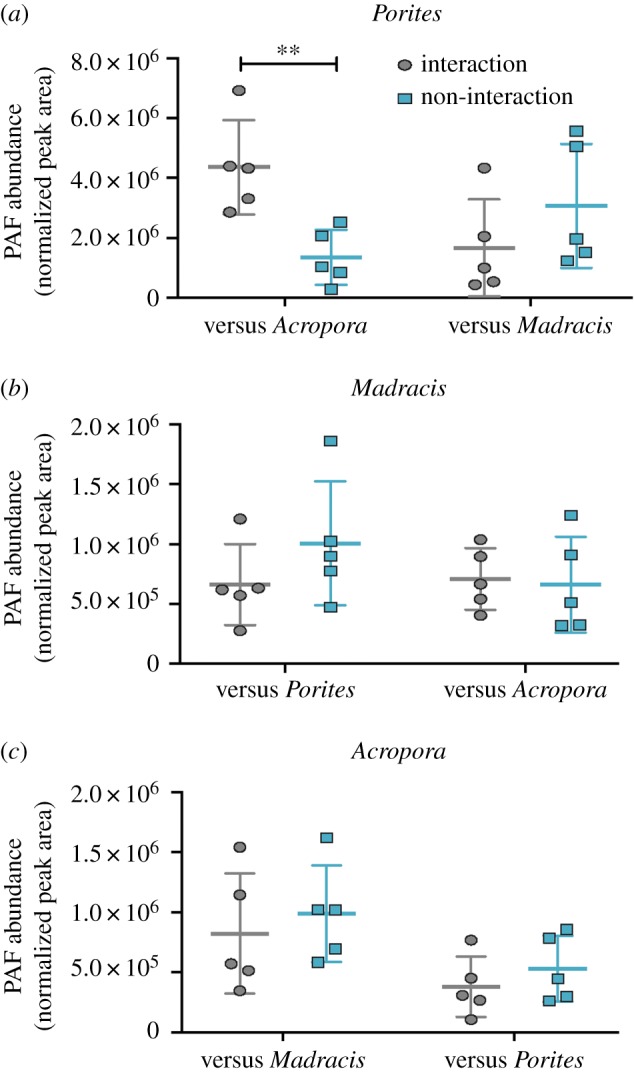
Platelet-activating factor (PAF) abundances (mean ± s.d.) in coral interactions. Normalized abundances of PAF in (*a*) *Porites porites* when interacting (grey ovals) or not interacting (teal rectangles) with *Acropora cervicornis* (*p* = 0.006; *n* = 5) and *Madracis mirabilis* (*p* = 0.27; *n* = 5); (*b*) *M. mirabilis* when interacting (grey ovals) or not interacting (teal rectangles) with *P. porites* (*p* = 0.25; *n* = 5) and *A. cervicornis* (*p* = 0.83; *n* = 5); and (*c*) in *A. cervicornis* when interacting (grey ovals) or not interacting (teal rectangles) with *P. porites* (*p* = 0.96; *n* = 5) and *M. mirabilis* (*p* = 0.59; *n* = 5). Asterisks denote the level of significance (**p < 0.01).

To then determine whether this phenomenon occurs in the natural environment, PAF was quantified in pairs of corals of different species growing in close proximity to one another on a reef. These pairs included *A. cervicornis* and *P. porites, A. cervicornis* and *M. mirabilis,* and *P. porites* and *M. mirabilis.* The only interaction in which mesenterial fighting was observed was when *A. cervicornis* attacked *P. porites.* During this interaction, PAF abundance in *P. porites* was higher in branches next to *A. cervicornis* compared to branches further away from the interaction site (figure 3*a*; *p* = 0.006; unpaired *t*-test). In *A. cervicornis*, PAF abundance remained stable when interacting with *P. porites* (figure 3*c*; *p* = 0.59), suggesting that PAF abundance does not change in the attacking coral, only in the coral being attacked. No significant difference in PAF production was observed when two coral species were adjacent to each other but not extruding their mesenteries (i.e. when no observed physical attack occurred): PAF abundance in *P. porites* was similar in branches regardless of whether they were near to or far from *M. mirabilis* branches (figure 3*a*; *p* = 0.27). Similarly, in *M. mirabilis*, PAF abundance remained unchanged when adjacent to *P. porites* (figure 3*b*; *p* = 0.25) and when adjacent to *A. cervicornis* (figure 3*b*; *p* = 0.83). Thus, the only interaction that resulted in increased PAF was when *P. porites* was physically attacked by *A. cervicornis*. This result suggests that PAF is associated with the tissue death response following direct contact by the mesenteries of a neighbouring coral colony.

### (e) Coral response through platelet-activating factor biosynthesis does not only occur at the point of physical contact

**Figure 4.**
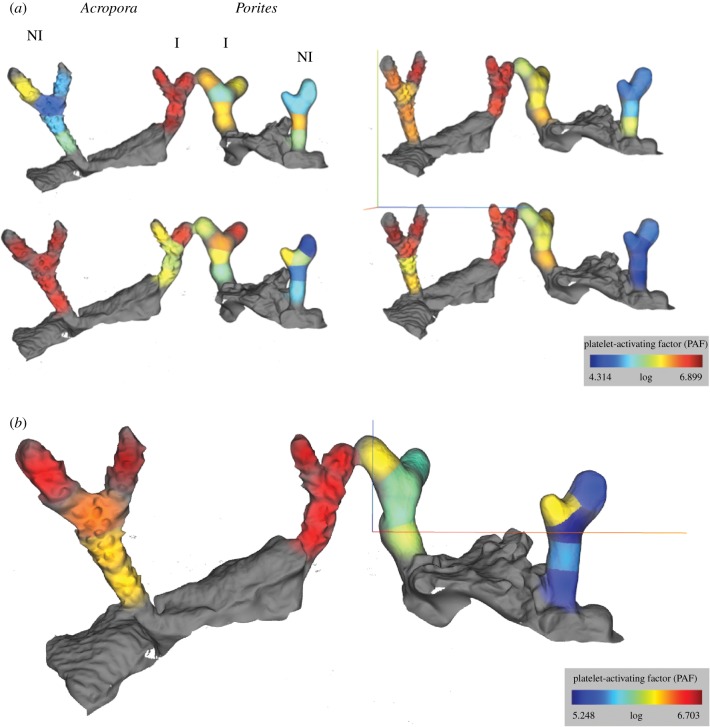
Molecular cartography of normalized platelet-activating factor (PAF) abundance (see intensity scale in colour) in *Acropora cervicornis* (left, labelled ‘*Acropora*’) with *Porites porites* (right, labelled *Porites*) when interacting (centre fragments, labelled ‘I’) or not interacting (fragment extremities, labelled ‘NI’; *n* = 4). (*a*) All four replicates; (*b*) the average of normalized PAF abundances from the replicates in (*a*).

Following attack by the mesenteries of a neighbour, we predicted that PAF production would spread from the point of competitive interactions throughout adjacent coral polyps within the same branch as a form of polyp-to-polyp communication, because coral polyps are assembled and network-connected in a complex colony. To test this, coral branches from eight corals were sampled in contiguous 1 cm sections. Molecular abundances were then mapped onto 3D models of each coral branch to visualize logarithmic PAF abundances along the branch (electronic supplementary material, movie S2 and figure S4). Cartographic projections showed that PAF was more abundant in *P. porites* interacting with *A. cervicornis* at the interaction zone (figure 4; *p* < 0.001) and throughout the entire branch (electronic supplementary material, figure S4; *p* < 0.001), suggesting that PAF biosynthesis extended beyond the zone of direct interaction. No significant difference was observed between *A. cervicornis* interacting with *P. porites* and *A. cervicornis* alone, as observed *de visu* earlier.

### (f) Presence of genes involved in platelet-activating factor synthesis cascade

Consistent with the abundance of PAF and Lyso-PAF in corals, the genes encoding for proteins involved in the synthesis of PAF were found in the genome of *Acropora digitifera*, a species in the same genus as the focal coral used in these studies. The presence of PLA2, Lyso-PAF acetyltransferase and PAF acetylhydrolase was recently reported in coral transcriptomes [10]. Many genes involved in PAF downstream signalling were also identified here, including: the PAF receptor (*E*-value: 1.09 × 10^−26^, 57% identity with the human protein sequence) and protein kinase C (*E*-value: 3.09 × 10^−45^, 87% identity with the human protein sequence). The presence of multiple genes in the mammalian PAF pathway suggests corals may synthesize and modify PAF during similar processes to those observed in mammals.

## 4. Discussion

As evidenced through our laboratory studies and field observations, increased PAF production occurs when corals experience environmentally- and organismal-induced physiological changes. Like humans, PAF production is higher in corals during active tissue growth and when exposed to increased levels of UVR. In corals, immune responses such as increased phenoloxidase activity and higher fluorescent protein levels have been observed in growing tissues [29,30] as well as during tissue regeneration following damage [20]. These findings, and the potential association between tissue growth and a PAF-associated immune response, suggest immune responses may be a common feature of tissue growth in corals. While tissue growth may be enhanced by PAF production, perhaps in concert with the activation of other immune processes, PAF can act as an immunosuppressant, as has been shown in mice exposed to UVR [14,22]. Thus, the increases in PAF in corals exposed to high UVR may lead to the depression of immune responses. Whether these processes activate or inhibit immune processes would be fertile subjects for future research aiming to better understand the coral immune system and cnidarians more generally.

While the increase in PAF observed in corals during growth and UVR stress is similar to responses in humans, PAF is also produced in situations specific to corals, specifically when under attack by the mesenterial filaments of a neighbouring coral colony. *Acropora* spp. corals were observed attacking neighbouring colonies with their mesenterial filaments in laboratory and natural settings. *Acropora* and other genera studied here have similar sensitivities to abiotic environmental changes (e.g. [31]), but the results presented here suggest divergent responses to ecologically relevant competitive interactions. When placed close to one another in the laboratory, *A. yongei* attacked *P. damicornis,* and when living in close proximity in the field, *A. cervicornis* attacked *P. porites*. By contrast, *A. cervicornis* living in close proximity to *M. mirabilis* and *P. porites* living in close proximity to *M. mirabilis* did not attack their neighbour. When *Acropora* colonies attacked neighbours with their mesenteries, PAF increased in the corals being attacked but did not change in the attacking *Acropora*. Similarly, PAF did not change in either species when neither species attacked the other with their mesenteries.

**Figure 5.**
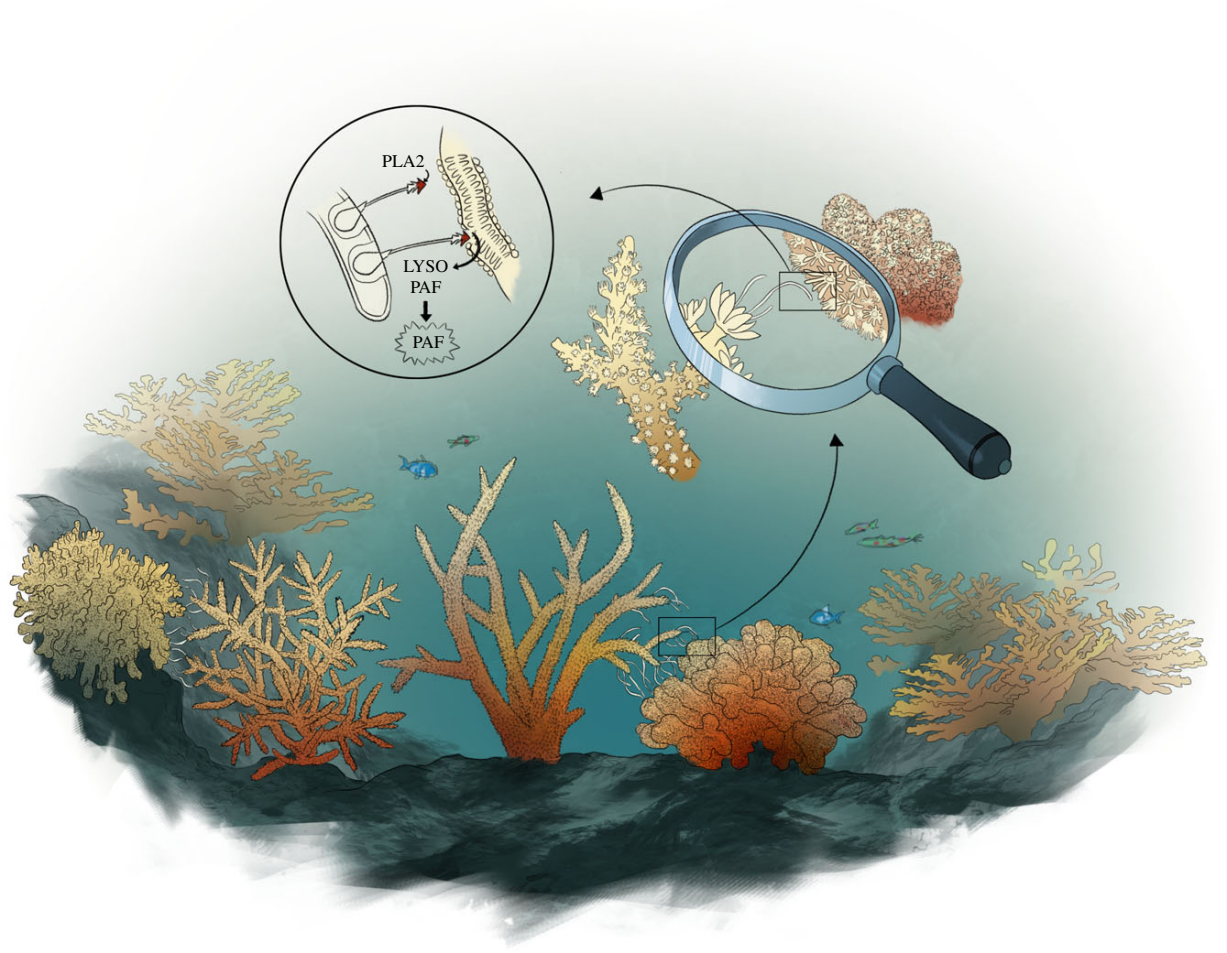
The proposed model of PAF production during coral competition. From the bottom rectangle going counterclockwise in a loop, the drawing represents a coral attacking (left) another coral (right) with its mesenterial filaments externalized from the mouth of the coral. In the last circular zoom, the harpoon-like nematocysts are projected from cnidocyte cells within the mesenterial filaments onto the attacked coral, penetrating the tissue. The tip of the harpoon contains venom with phospholipase A2 that is injected into the tissue of the attacked coral, stimulating the conversion of the attacked coral's phosphatidylcholine into Lyso-PAF and then PAF, leading to inflammation in the attacked coral tissue. Illustration by I.G.A.

The tentacle-like mesenteries contain large numbers of venom-containing nematocysts that induce inflammatory reactions in other organisms, including humans [32]. One of the most pro-inflammatory enzymes found in many types of venom of organisms (e.g. cobra, honeybee) is PLA2, which initiates the production of PAF. PLA2 is abundant in stony corals, particularly in their mesenteries and other attacking structures [25]. In addition, PLA2 is highly expressed and PAF is abundant at interaction zones between corals in the Southern Line Islands [10]. Thus, increased PLA2 activity probably induces the increase in PAF production observed during mesenterial attack, revealing a molecular response underlying the response of corals to mesenterial attack. We propose that by trafficking PLA2 to a neighbouring coral via its mesenteries, attacking corals stimulate overwhelming production of PAF in the attacked coral (figure 5), leading to acute inflammation and severe tissue damage in the latter, exploiting this conserved process and eliminating the competitor. For corals with similar growth strategies and energy requirements, the production of defensive molecules is particularly important in determining their success on the reef (as opposed to overgrowth or shading neighbours). While previous work has mostly focused on the direct passage of harmful molecules to a competitor [33,34], here we highlight how overstimulation of conserved processes in the attacked coral can be a potent force in determining interaction outcomes, thereby influencing the composition of coral assemblages in reef ecosystems.

## Supplementary Material

Electronic Supplementary Material

## Data accessibility

Data are archived in the Dryad Digital Repository at: http://dx.doi.org/10.5061/dryad.ct17vn7 [35].
