# Supplementary material for: Before platelets: the production of platelet-activating factor during growth and stress in a basal marine organism
Source: Proc Biol Sci. 2018 Aug 15;285(1884):20181307. doi: 10.1098/rspb.2018.1307 (PMC6111180; doi:10.1098/rspb.2018.1307)
Supplement: Electronic Supplementary Material [file rspb20181307supp1.docx]

**Before platelets: the production of platelet activating factor during growth and stress in a basal marine organism**

Ines Galtier d’Auriac, Robert A. Quinn, Heather Maughan, Louis-Felix Nothias, Mark Little, Clifford A. Kapono, Ana Cobian, Brandon T. Reyes, Kevin Green, Steven D. Quistad, Matthieu Leray, Jennifer E. Smith, Pieter C. Dorrestein, Forest Rohwer, Dimitri D. Deheyn, Aaron C. Hartmann

**ELECTRONIC SUPPLEMENTARY MATERIAL**

Figure S1—S6

Movies S1—S2


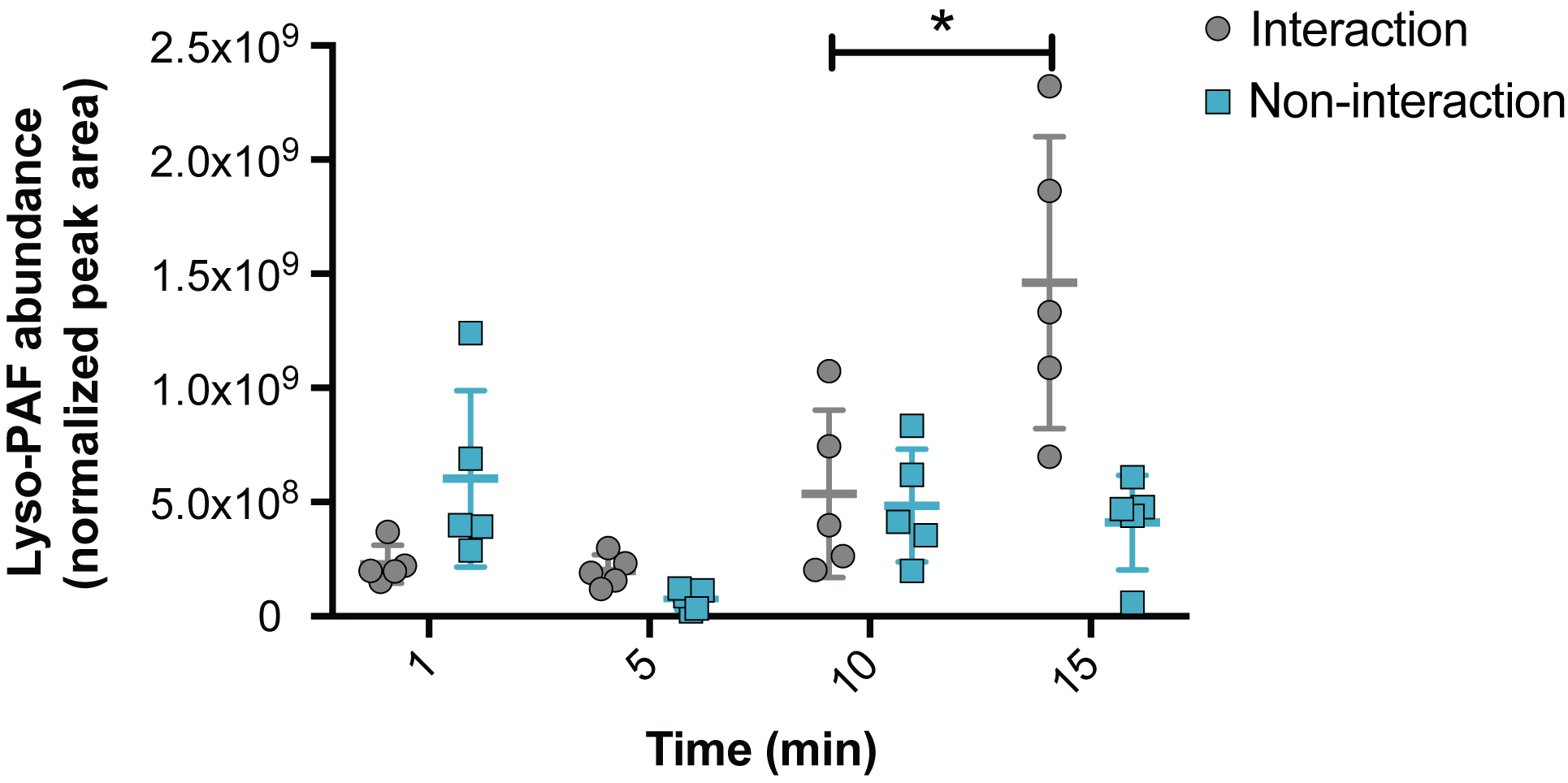


**Figure S1.** Normalized abundance (mean ± SD) of Lyso-PAF in *Pocillopora damicornis* after 1, 5, 10 and 15 minutes of interacting with *Acropora yongei* (grey circles; *p* < 0.001, one-way ANOVA test, *p* = 0.02) and when not interacting (teal squares; *p* = 0.069).


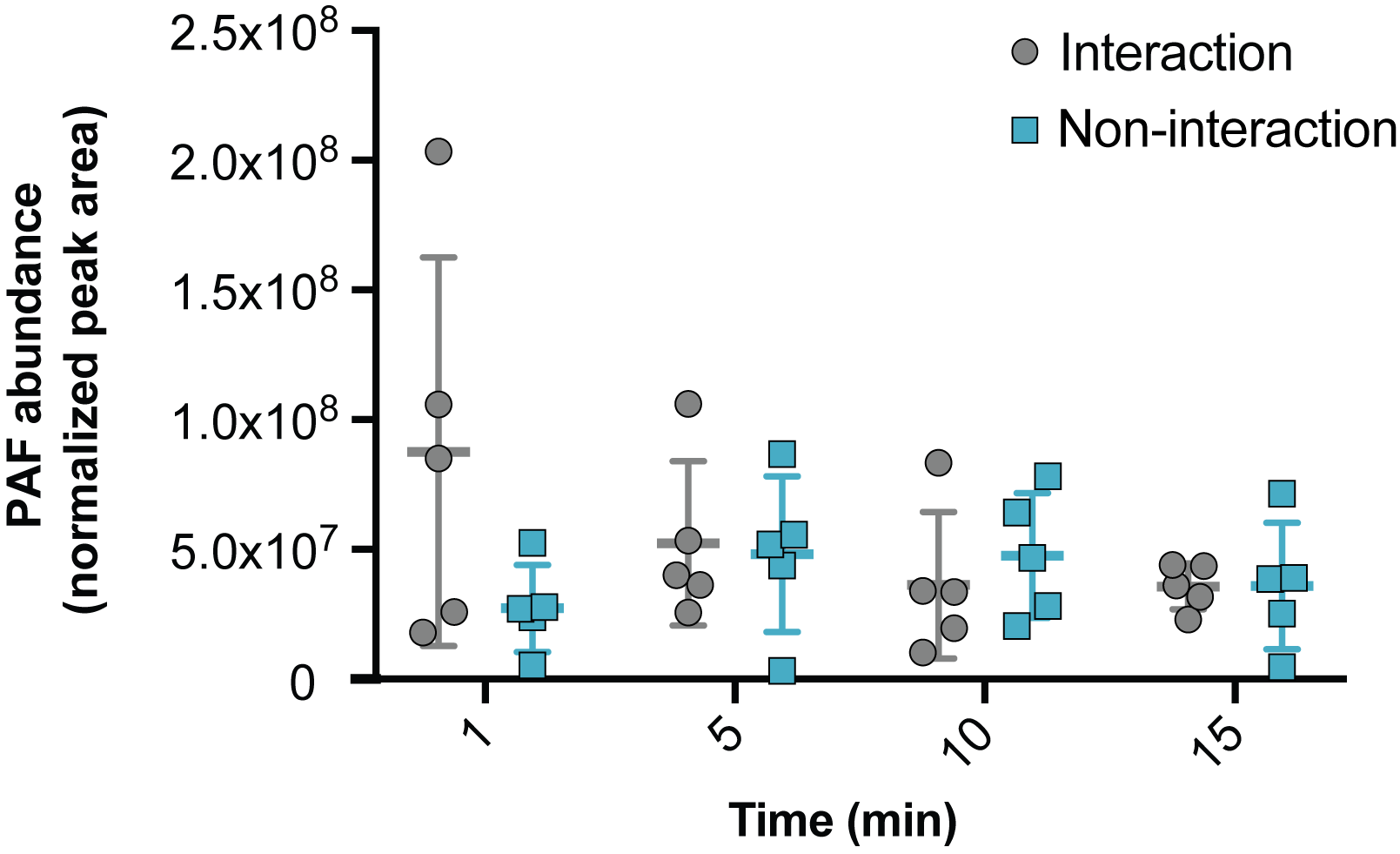


**Figure S2.** Normalized abundance (mean ± SD) of PAF in *Acropora yongei* after 1, 5, 10 and 15 minutes of interaction (grey circles) with *Pocillopora damicornis* (*p* = 0.2311; one-way ANOVA test) and when not interacting (teal squares) with *P. damicornis*.


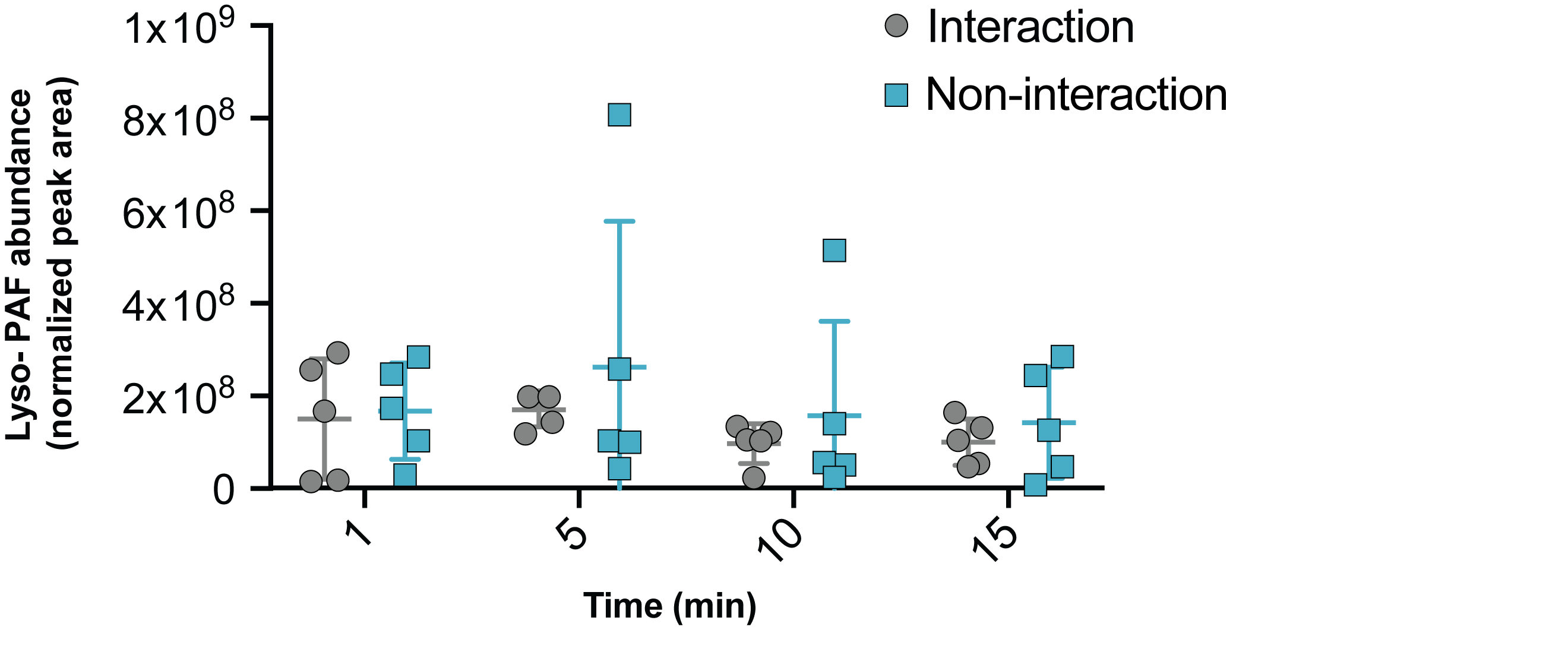


**Figure S3.** Normalized abundance (mean ± SD) of Lyso-PAF in *Acropora yongei* after 1, 5, 10 and 15 minutes of interaction (grey circles) with *Pocillopora damicornis* (*p* = 0.3535, one-way ANOVA test) and when not interacting (teal squares) with *P. damicornis*.


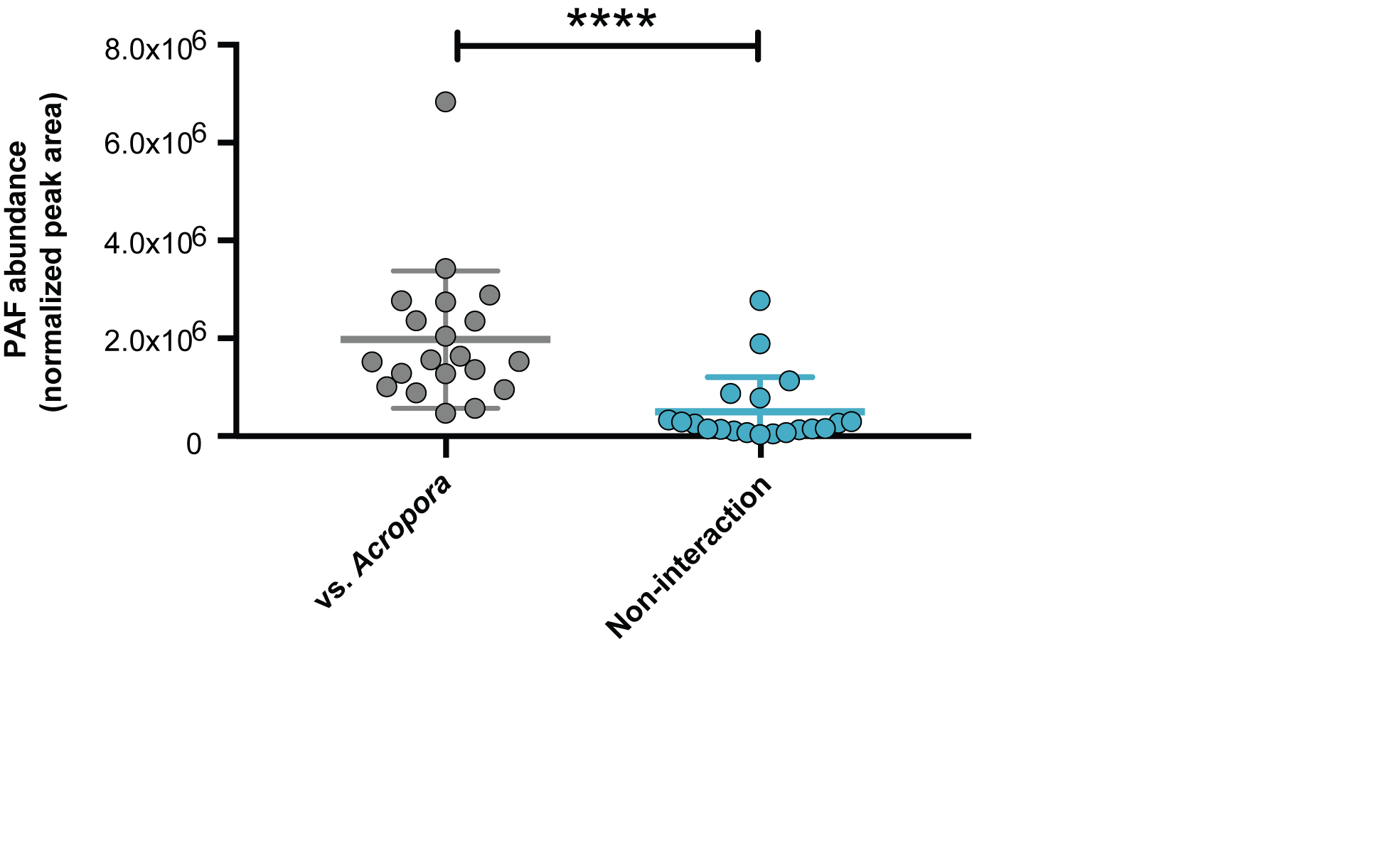


**Figure S4.** Normalized abundances (mean ± SD) of PAF in *Porites porites* during interaction (grey circles) with *Acropora cervicornis* (*p* < 0.001) and when not interacting (teal circles) with *A. cervicornis* in Bocas del Toro, Panama (n = 20).


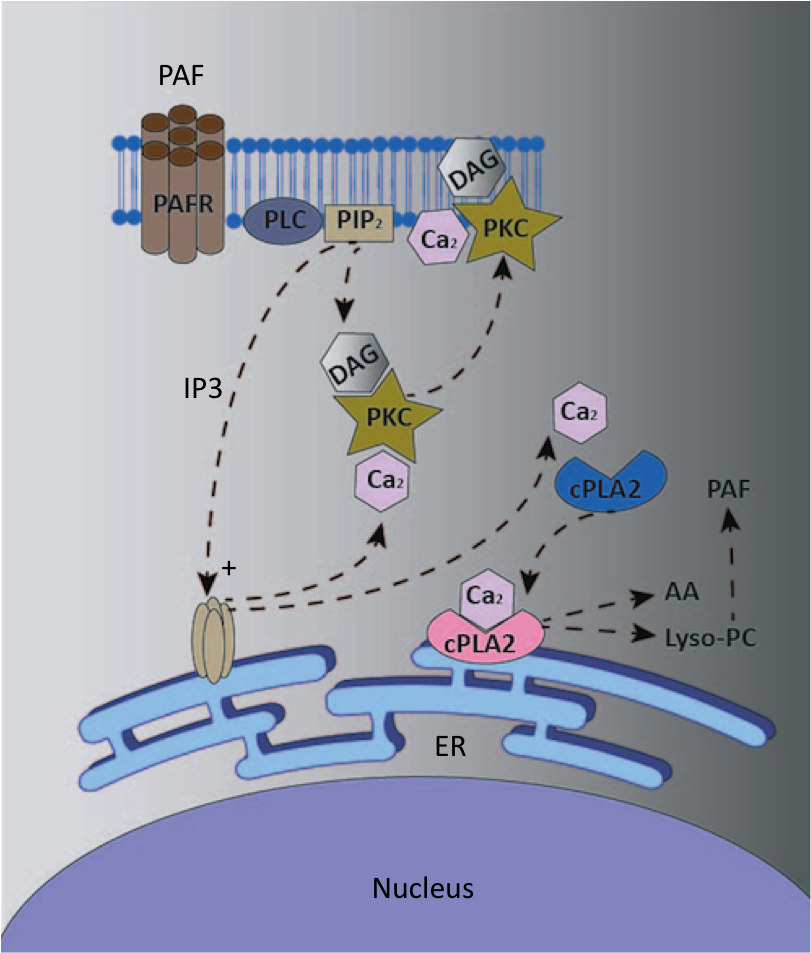


**Figure S5.** Schematic representation of PAF molecular mode of action. PAF binds to its receptor (PAFR) which activates the breakdown of phosphoinositol biphosphate (PIP2) by phospholipase C (PLC), which then releases inostitol triphosphate (IP3) and diacyl glycerol (DAG). IP3 activates the endoplasmic reticulum (ER) Ca^2+^ pump. DAG and Ca^2+^ activates protein kinase C (PKC) and Ca^2+^ also activates cytosolic phospholipase A2 (cPLA2). cPLA2 catalyzes the biosynthesis of arachidonic acid (AA) and Lyso-PC (precursor of PAF) from ER phosphatidylcholine.

**
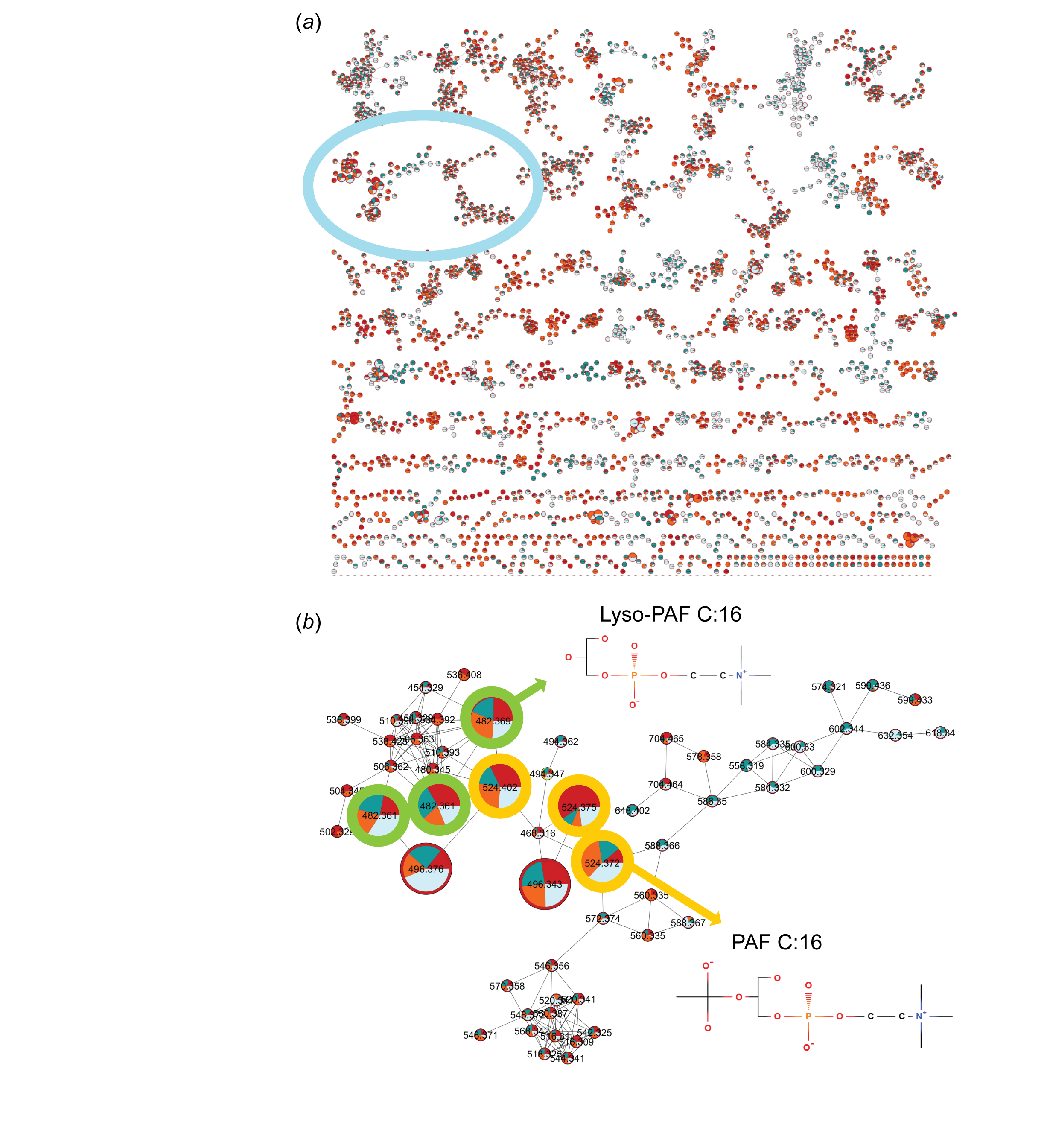
**

**Figure S6.** Molecular network from *Acropora* *cervicornis* and *Porites* *porites* interactions *in situ* (*a*) where each node represents a compound connected by a mass translation, red represents *A. cervicornis* not interacting, orange is *A. cervicornis* interacting with *P. porites*, blue is *P. porites* not interacting and light blue is *P. porites* interacting with *A. cervicornis*. (*b*) Molecular network of PAF and Lyso-PAF from *A. cervicornis* and *P. porites* interactions in Panama.

**Movie S1.** A stop motion video of an *Acropora yongei* coral colony attacking a *Pocillopora damicornis* colony, made over 72 hours of interaction, and available at https://www.youtube.com/watch?v=HYD5XJR4CVA. *A. yongei* senses the neighboring coral and extrudes its mesenteries onto the *P. damicornis,* resulting in tissue loss in the latter.

**Movie S2.** A rotating image of a 3D model of *Acropora cervicornis* coral interacting with *Porites porites,* including the PAF molecular cartography, available at: https://ili.embl.de/?https://github.com/lfnothias/coral_PAF/raw/master/3D_Mol_Cartography/Coral_PAF_3D_model.stl;https://github.com/lfnothias/coral_PAF/raw/master/3D_Mol_Cartography/Coral_PAF_ili_table.csv;https://github.com/lfnothias/coral_PAF/raw/master/3D_Mol_Cartography/Platelet_Activating_Factor_(PAF)_C-16(ID_%20116).json.
